# Long-term outcomes and patient profiles following intensity-modulated radio-chemotherapy for nasopharyngeal cancer in a nonendemic region

**DOI:** 10.3389/fonc.2026.1724193

**Published:** 2026-01-27

**Authors:** Tatiana Dragan, Ibrahim Chiairi, Maurine Salmon, Yassine Lalami, Antoine Digonnet, Samuel Lipski, Dirk Van Gestel

**Affiliations:** 1 Department of Radiation Oncology (Head and Neck Unit), Institut Jules Bordet, Université Libre de Bruxelles, Brussels, Belgium; 2 Department of Radiation Oncolgy, AZORG Hospital, affiliated with KU Leuven, Aalst, Belgium; 3 Data Centre, Institut Jules Bordet, Université Libre de Bruxelles, Brussels, Belgium; 4 Medical Oncology Clinic, Institut Jules Bordet, Université Libre de Bruxelles, Brussels, Belgium; 5 Department of Head and Neck Surgery, Institut Jules Bordet, Université Libre de Bruxelles, Brussels, Belgium; 6 Université Libre de Bruxelles, Brussels, Belgium

**Keywords:** Epstein-Barr virus, IMRT (intensity modulated radiation therapy), nasopharyngeal carcinoma, non endemic area, outcomes

## Abstract

**Introduction:**

Nasopharyngeal carcinoma (NPC) is rare in non-endemic regions but poses unique clinical challenges, especially among ethnically diverse populations. This study aimed to evaluate the characteristics, treatment outcomes, and toxicity profiles of NPC patients treated with definitive intensity-modulated radio-chemotherapy (IMRT-CRT) in a non-endemic European center.

**Methods:**

We conducted a retrospective analysis of 82 consecutive patients with histologically confirmed NPC treated at Institut Jules Bordet between 2012 and 2024. Patient, tumor, treatment characteristics, survival outcomes, and treatment-related toxicity were evaluated. Survival endpoints were calculated using the Kaplan-Meier method and multivariate Cox regression.

**Results:**

The median follow-up was 35 months. Most patients were male (77%), of North African descent (66%), and had non-keratinizing carcinoma (96%). Advanced disease (stage III/IV) was present in 72%, and 78% of tested tumors were EBV-positive. Induction chemotherapy (ICT) and concurrent chemotherapy (CCT) were administered in 32% of patients. At 5 years, actuarial estimates for overall survival (OS), progression-free survival (PFS), locoregional disease-free survival (LRDFS), recurrence-free survival (RFS), and distant disease-free survival (DFS) were 73%, 79%, 86%, 87%, and 87%, respectively. Higher T stage, treatment-related weight loss, positive nodal findings on 3-month FDG-PET-CT, and disease recurrence were associated with worse OS. Acute and late toxicities were generally manageable; however, xerostomia, hypothyroidism, ototoxicity, and weight loss were prevalent.

**Conclusions:**

Despite being conducted in a non-endemic region, this study revealed NPC characteristics and outcomes resembling those in endemic populations, likely due to patient demographics. Early metabolic response and nutritional status emerged as critical prognostic factors, highlighting the need for individualized treatment and supportive care strategies in NPC management.

## Introduction

The incidence of nasopharyngeal carcinoma (NPC) exhibits considerable geographic variation with global age-standardized incidence rates of 2.2 per 100 000 in men and 0.8 in women. It has a unique geographic distribution, endemic in Southeast Asia (15–50/100 000) and less common (0.2–0.5/100 000) in Western countries (1). The World Health Organization (WHO) classifies three primary histological subtypes: keratinizing squamous cell carcinoma (WHO type 1), differentiated nonkeratinizing carcinoma (WHO type 2a) and undifferentiated nonkeratinizing carcinoma (WHO type 2b). In regions where the disease is endemic, nearly all cases are of the undifferentiated nonkeratinizing type, whereas the keratinizing subtype is more prevalent in Western countries (2). In Asian populations, undifferentiated carcinoma associated with Epstein-Barr virus (EBV) is the predominant subtype of NPC (3, 4). In contrast, in non-endemic regions, a significant number of NPC cases are EBV-negative and present as keratinizing or differentiated non-keratinizing carcinoma. These subtypes are linked to poorer overall survival (OS), largely due to lower rates of locoregional and distant disease control (3–5). Nasopharyngeal cancer also shows familial clustering, suggesting that genetic susceptibility, along with EBV infection and environmental factors, collectively influence disease risk. For example, consistent evidence indicates that consumption of salty foods increases NPC risk by two to three times (6, 7). Tobacco smoking is another significant risk factor, elevating the risk by two to six times, while the association between alcohol consumption and NPC remains unclear (7). EBV is classified as a Group 1 carcinogen by the International Agency for Research on Cancer with respect to NPC, indicating that there is sufficient evidence of its carcinogenicity in humans (8, 9). EBV can be detected by in situ hybridization through the presence of EBV-encoded RNAs in NPC tissue. Latent EBV is consistently found in high-grade dysplasia and NPC cells, but not in normal epithelium or low-grade dysplasia (10).

Since the beginning, Radiotherapy (RT) stands as the primary treatment modality for NPC. The advent of intensity-modulated RT (IMRT) has demonstrated notable enhancements in both locoregional control and OS when compared to three-dimensional conformal RT and two dimensional RT (11, 12). Furthermore, a pooled analysis by Lee et al. (13), comparing conventional and accelerated RT, with or without chemotherapy (CT; both concurrent and adjuvant), revealed variables such as radiation dose, fractionation, and the number of CT cycles (both concurrent and adjuvant) to significantly impact both survival and toxicity.

Stage I NPC is effectively managed with definitive RT alone whereas patients with stage II benefit from concurrent chemoradiotherapy (CRT). Stage III and IVA NPC is treated with CRT, with cisplatin as the standard concurrent agent improving OS and disease control (14). The typical regimen is cisplatin 100 mg/m² every three weeks alongside RT though weekly cisplatin (40 mg/m²) also shows OS benefit (15, 16). A cumulative cisplatine dose exceeding 200 mg/m² is recommended (17). In locally advanced stages of NPC, distant metastasis is a major cause of failure, so systemic treatment intensification is necessary. A phase III trial compared ICT using cisplatin and gemcitabine followed by concurrent CRT against CRT alone in patients with stage III/IVB NPC (as per AJCC 7th edition). The trial found ICT to improve recurrence-free survival (RFS), OS, and distant RFS, although it caused higher acute but not late toxicities (18). Notably, 96.7% of patients assigned to the ICT group completed the three cycles of cisplatin/gemcitabine, and 92% received at least two cycles of cisplatin 100 mg/m² alongside RT. This study excluded patients with T3–4 N0 disease. A recent meta-analysis demonstrated that ICT significantly improves both OS and progression-free survival in patients with locally advanced NPC, supporting its use as a standard component of treatment in this setting (19).

The role of adjuvant CT in NPC has been investigated in the past; however, its use has been limited due to concerns regarding overall toxicity and treatment tolerance. Adjuvant CT often proves difficult to complete, with only about 60% of patients able to finish the planned treatment cycles, and approximately half requiring dose reductions (20). Despite these challenges, recent evidence from two phase III randomized trials supports the use of adjuvant capecitabine, administered either at a standard dose or in a metronomic regimen (21, 22). In patients with high-risk locoregionally advanced NPC, the addition of capecitabine following CRT has been associated with a significant improvement in failure-free survival compared to observation alone.

Although extensively studied by research groups in endemic regions, data on nasopharyngeal carcinoma outcomes in non-endemic areas remain limited. In this single center retrospective study we will assess the epidemioclinical characteristics, survival outcomes, as well as acute and late toxicity in NPC patients undergoing CRT in a non-endemic area.

## Materials and methods

### Study design and patient population

This retrospective single-center study encompasses patients with pathologicaly proven NPC, consecutively treated with IMRT at Institut Jules Bordet between January 2012 and February 2024. Any CT was administered either within the same center or at an affiliated hospital (CHU Saint-Pierre, Hospital Erasme, Hospitals IRIS Sud, CHU Brugmann). Comprehensive data retrieval involved a thorough review of electronic and written case notes, focusing on patients meeting the eligibility criteria. Staging information adhered to the American Joint Committee on Cancer eighth edition, while histological classification followed the WHO criteria, distinguishing between keratinizing and non- keratinizing, differentiated, or undifferentiated subtypes. Inclusion criteria were age 18 years or older; histologically confirmed keratinizing SCC, nonkeratinizing differentiated carcinoma, or undifferentiated carcinoma of the nasopharynx; and treatment with radical CRT or RT alone. Exclusion criteria included a prior history of irradiation and the use of RT dose/fractionation schedules other than 70 Gy in 35 fractions or 69.12 Gy in 32 fractions.

### Treatment

Patient treatment adhered to institution-specific protocols, with CT regimens tailored to each facility’s guidelines. Typically, RT was given in a supine position using a five-point thermoplastic shell. Planning was done on computed tomography scans with 2–3 mm slices, and intravenous contrast was administered when the renal function allowed for it and there was no history of allergic reactions to contrast. Diagnostic magnetic resonance (MRI) and positron emission tomography-computed tomography (FDG-PET-CT) imaging was carried out for all patients. Target volumes were determined based on clinical and endoscopic examination and diagnostic imaging. A rigid co-registration of the region of interest was performed with FDG-PET-CT and MRI sequences (including T1 gadolinium and T2 sequences). Since 2017, all HNC contours in our department have been peer-reviewed by a multidisciplinary team of HNC-dedicated radiation oncologists, radiologists, and nuclear medicine physicians to ensure consistent definition of the primary and nodal gross tumor volumes (GTVp and GTVn) (23).

The high-risk clinical target volume (CTV) was defined as the GTV plus a 0.5-cm margin. The low-risk CTV was delineated based on the estimated risk of microscopic spread, including the whole nasopharynx and taking into account the anatomical site and stage of the primary NPC, in accordance with the recommendations of RTOG (24) and since 2017, we have followed the international guidelines of Lee et al., 2017 (25). It also included regional elective lymph nodes, depending on nodal status (24, 25). No intermediate-risk CTV was defined; thus, target delineation was limited to two CTVs only (high-risk and low-risk). Following induction chemotherapy, response was assessed using a combination of radiologic (MRI and FDG-PET-CT) and endoscopic evaluations, following standard criteria for NPC. The target delineation has been based on the pre-chemotherapy GTV, with margins preserved for involved structures such as bone, the skull base, and the sinuses. In case of large tumours with good result of induction therapy, the CTV was modified to spare critical normal tissue. For involved nodes with visible soft tissue extracapsular extension, the GTVn was defined by the pre-induction chemotherapy involved region. CTV to planning target volume (PTV) margins were 5 mm. Two dose levels were used, the high-dose PTV was prescribed 70/69.12 Gy, and the elective-dose PTV was prescribed 56 Gy. Our institution consistently employed a radiation dose of 70 Gy delivered in 35 fractions until the end of 2018. Subsequently, we transitioned to a more hypofractionated regimen of 69.12 Gy administered in 32 fractions, with treatment sessions scheduled from Monday to Friday over a duration of 6.5–7 weeks. Radiation dose prescription was performed in accordance with ICRU guidelines (26) ensuring that the prescribed dose corresponded to the dose delivered to the target volume while maintaining appropriate dose homogeneity and respecting dose constraints to surrounding organs at risk. All patients were treated with volumetric modulated arc therapy (VMAT) with daily-image guidance using cone-beam CT (CBCT). The addition of induction, concurrent and/or adjuvant CT was decided upon in the multidisciplinary team discusion.

### Response evaluation and follow-up

Response evaluation was performed using imaging modalities, MRI, CT scanner, and FDG-PET-CT, according to institutional practice. MRI was routinely performed 2 months after completion of RT to assess local and nodal response, while FDG-PET-CT was used at 3 months post-RT for metabolic response evaluation. Cases with equivocal imaging findings were reviewed in a multidisciplinary tumor board, and when indicated, repeat FDG-PET-CT with or without MRI was performed at 6 months before further management decisions, in line with the approach described by Mehanna et al. (27). All patients were clinically assessed, weekly during RT and 1 month after the treatment. Subsequently, the follow up was every 2–3 months for the first 3 years, every 3–6 moths for the 4th and 5th years and then yearly further on. Local, regional, or distant relapse was determined through a comprehensive evaluation incorporating clinical assessment, radiological imaging, and histological confirmation.

### Statistical analysis

Descriptive analyses were conducted. Summary statistics, the mean, the standard deviation, median and interval inter-quartile (IQR) were computed for continuous variables whereas counts and percentages were computed for categorical variables. Comparison test was used to compare weight loss between patient orally fed and fed with a feeding tube. A Wilcoxon rank sum test was used in case of the rejection of hypothesis of normality between the groups. The study employed several endpoints for assessment, including OS and cancer related death, PFS, LDFS, RDFS and DDFS. Survival analysis were performed excluding 5 patients with metastatic disease at diagnosis. The outcome was calculated from the time of first treatment until death from any cause (OS) or cancer related death. If the patient was still alive at last follow-up, the patient was censored at last follow-up. Descriptive survival analyses were conducted utilizing the Kaplan-Meier method. The median survival timewas computed as well as the median follow-up, median time to first recurrence (local, regional and distant), median time to local relapse, median time to regional relapse and median time to distant failure. A univariate survival analysis was primarily conducted. The Cox regression model was used for OS whereas competing risk analysis was used with a Fine and Gray model for cancer related death analyses with death from other cause included as competing event. Significant variables at the threshold p < 0.05 were included in a multivariate analysis with a stepwise backward forward method.

### Ethics approval

The study protocol was reviewed and approved by the Ethics Committee of Institut Jules Bordet, Brussels, Belgium, CE 3765, on January 18, 2024.

## Results

### Patient, disease and treatment characteristics

In total, 82 patients were included in this study. The median duration of follow-up was 35 months (IQR = 41 months). Median age was 48.5 years at time of diagnosis. Seventy-seven percent were men and 23% women. Seven percent had a family history of NCP. The majority of patients (66%) were of North African ethnicity, 16% were of Western European descent, and 10% were of Southern European descent. Histologically, most tumors were non-keratinizing (96%), including 14% differentiated, 40% undifferentiated, and 42% with unknown differentiation, while keratinizing carcinoma accounted for 4% of cases. Tumor EBV-status was determined in 85% of patients, with 78% of the tested cases being EBV-positive. Seventy two percent of patients had stage III or IV disease; 12% and 24% of patients had T3 and T4 disease, respectively, whereas 59% of patients had N2/3 disease. AJCC 8th edition stage distribution was I–II: 58%, III–IVA: 48%, and IVB: 5%. The most common initial presenting symptom was a neck lump, observed in 31% of cases. Hypoacousia and nasal congestion were reported in 17% of cases each, while ear pain was noted in 14% of cases. Fifty-five patients (67%) were WHO grade 1, and 27 patients (33%) were WHO grade >1 (**Tables 1**, **2**).

**Table 1:** Patient and disease characteristics.

Characterisic	Number of patients (% or IQR)
Cohort	82 (100%)
Median duration of follow-up	150 weeks (179)
Age	
Median (IQR)	49years (17 years)
Gender	
Male	63 (77%)
Female	19 (23%)
Performans status	
WHO = 1	55 (67%)
WHO > 1	27 (33%)
Ethnicity	
North African	54 (66%)
Western Europe	13 (16%)
South europe	8 (10%)
Asian	3 (4%)
Eastern Europe	2 (2%)
Latin American	2 (2%)
T stage	
T0	1 (1%)
T1	33 (41%)
T2	18 (22%)
T3	10 (12%)
T4	20 (24%)
N stage	
N0	11 (13%)
N1	23 (28%)
N2	41 (50%)
N3	7 (9%)
M stage	
M0	77 (94%)
M1	5 (6%)
AJCC-stage (8th edition)	
I	5 (5%)
II	19 (23%)
III	31 (38%)
IVA	23 (28%)
IVB	5 (6%)
Histology	
Keratinising carcinoma (WHO type 1)	3 (4%)
Non-keratinising differentiated carinoma (WHO type 2a)	12 (14%)
Non-keratinising_undifferentiated carcinoma (WHO type 2b)	33 (40%)
Non-keratinising,differentiation unknown	34 (42%)
Smoking	
Never	35 (43%)
Former	18 (22%)
Current	29 (35%)
Alcohol	
None	49 (60%)
Former	24 (29%)
Current	9 (11%)
EBV status	
Positive	64 (78%)
Negative	6 (7%)
Unknown	12 (15%)
First symptom	
Lump (neck)	25 (32%)
Hypoacousia	14 (17%)
Nasal congestion	14 (17%)
Ear pain	12 (14%)
Headache	5 (6%)
Bleeding nose	4 (5%)
Diplopia	3 (4%)
Cranial nerve paralysis	2 (2%)
Trouble breathing	1 (1%)
Unknown	2 (2%)

AJCC, American Joint Committee on Cancer; CI, confidence interval;EBV, Epstein Barr virus; SCC, squamous cell carcinoma. IQR, intequartile range, WHO, World Health Organization

**Table 2:** Treatment characteristics.

Characteristics	Number of patients (% or IQR)
Induction chemotherapy	
No	56 (68%)
Yes	26 (32%)
Median age	43 years
Median OTT	44 days (8 days)
Induction regimen	
Cisplatinum/Gemcitabine	18 (22%)
TPF	6 (7%)
Cisplatinum/5FU None	2 (2%) 56 (69%)
Induction chemotherapy cycles	26 pts
1	0
2	3 (12%)
3	22 (84%)
4	1 (4%)
Disease stage and induction chemotherapy 26 pts	
I	0
II	4 (15% of stage II pts)
III	8 (31% of stage III pts)
IV	14 (54% of stage IV pts)
Responds evaluation method	
CT	4 (14%)
MRI	19 (63%)
PETCT	4 (13%)
Unknown	3 (10%)
Radiological response after induction chemotherapy	
Partial response	21 (81%)
Complete response	2 (8%)
Unknown	3 (11%)
Concurrent chemotherapy	
No	7 (9%)
Yes	75 (91%)
Median age	48 years (18)
Concurrent regimen	
Cisplatinum - 3 weekly	55 (67%)
Cisplatinum - weekly	13 (16%)
Carboplatinum weekly	6 (7%)
Cisplatinum + 5FU	1 (1%)
None	7 (9%)
5FU, 5-Flurouracil	
Radiotherapy	
Median time between simulation and first fraction	15 days
Median OTT 32 fractions	44 days
Median OTT 35 fractions	49 days
Fractionation	
35 fractions	40 (49%)
32 fractions	41 (50%)
Other (early stopped)	1 (1%)

Overall, 32% of patients received ICT. The median age of patients receiving ICT was 43 years (range 14-73). ICT was administered in 17%, 32% and 45% of patients with stage II, III and IV diseases, respectively. The most commonly used ICT regimen was Cisplatin/Gemcitabine, administered in 69% of cases, followed by docetaxel/cisplatin/fluorouracil (TPF) in 23% of cases, and Cisplatin + 5-FU in 8% of cases. Ninety-one percent of patients received CT concurrent with RT. The median age of patients treated with CRT was 48 years (range 39-57). Concurrent CT was administered in 87%, 100% and 96% of patients with stage II, III and IV diseases, respectively. The most commonly used CRT regimen was Cisplatinum 100mg/m3, administered in 74% of cases, followed by Cisplatinum weekly in 17% of cases, and Carboplatinum in 8% of cases. Only 6% of patients received adjuvant CT. The median age of patients treated with adjuvant CT was 42 years (range 35-50). Adjuvant CT was administered in 16% and 5.5% of patients with stage III and IV disease, respectively. A comprehensive table detailing the number of cycles for the chemotherapy regimens is presented in Annex 1. All but one patient completed the planned course of RT due to non-compliance to the treatment. The median overall RT duration was 47 days (range 43-62). The median time from simulation to the first fraction was 15 days (range: 5- 27). Patient, disease and treatment characteristics are summarised in **Tables 1**, **2**.

### Response evaluation

At 2 months post-RT, MRI assessment demonstrated a local complete response in 70% of patients, while 30% demonstrated an incomplete response. Regarding the lymph nodes, 63% of patients achieved a complete response on MRI, 35% had an incomplete response, and 1 patient showed disease progression. At 3 months post-RT, response assessment with FDG-PET-CT revealed a local complete response rate of 88%, an incomplete response in 10%, and disease progression in one patient. The locoregional nodal response rate was similarly high, with 88% achieving a complete response and 12% showing an incomplete nodal response.

#### Toxicity

Maximum short (within 90 days post-RT) and long (beyond 90 days post-RT) term toxicity were reported according to the CTCAE criteria. The median time for assessment of the short-term toxicity was 32 days (IQR = 11), while late-term toxicity was assessed at a median of 117 days post-treatment. Within 90 days of ending RT, 5% of patients experienced grade 2 or higher dermatitis, with no cases reported 90 days after the ending of radiation. Oropharyngeal mucositis was observed in most patients within 90 days of ending RT, with 20% exhibiting grade 2 and 11% presenting grade 3 mucositis, which disappeared in the long term. Grade 2 dysphagia was present in 25% of patients, and 5% had grade 3 dysphagia. Xerostomia was the most frequent side effect. Within 90 days of ending RT, grade 2 xerostomia occurred in 70% of patients and grade 3 in 2%. Over time, this evolved to 49% with grade 2 and 5% with grade 3 xerostomia. Tinnitus and hearing loss were reported by 26% and 14% of patients, and increased to 43% and 21% at short and long term, respectively. However, no hearing test was taken before starting treatment, so we can only reflect the subjective data of the patients. Short term vision problems were observed in 15% of patients and remained stable at 14% after 90 days of ending RT. On the long term, 13% of patients exhibited the Lhermitte’s sign, 4% had a reduction in function of one cranial nerve, and another 4% had a complete loss of function of one cranial nerve. Clinical hypothyroidism requiring substitution therapy, developed in 47% of irradiated patients.

**Figure 1:**
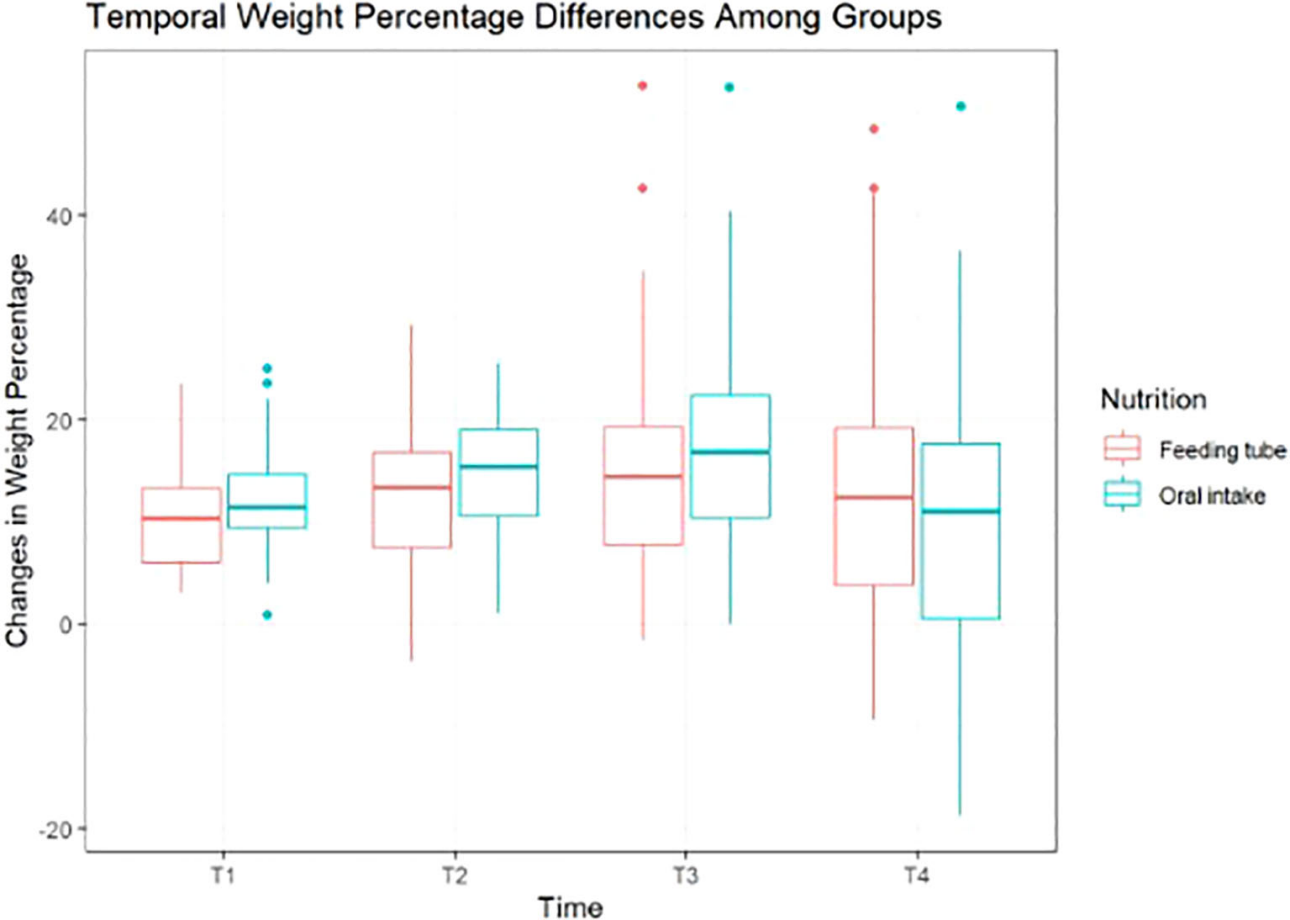
Temporal changes in weight percentage among groups by nutrition type. T1: Last day of radiotherapy, T2: short-term follow-up, within 90 days of the last fraction, median days 32; T3: medium-term follow-up, 90 days post-radiotherapy, median 117 days); T4: final follow-up, median 183 weeks, or approximately 3.5 years.

During radiotherapy (RT), 57% of patients maintained oral intake, 39% required enteral tube feeding, and 4% received short-term parenteral nutrition. At the end of RT, mean weight loss was 11%, predominantly grade 2, and was slightly higher in patients with oral intake than in tube-fed patients (median 11.5% vs. 10.5%). At short-term follow-up (≤90 days post-RT; median 32 days), median weight loss increased to 14.5%, with 61% of patients experiencing grade 2 and 13% grade 3 weight loss; feeding tubes remained in place in 21% of patients. Weight loss remained greater among patients maintaining oral intake (15.5%) compared with tube-fed patients (13%), with similar proportions of grade 3 loss (13%). At medium-term follow-up (≥90 days post-RT; median 117 days), median weight loss reached 16%, with grade 3 weight loss observed in 35% of patients, again higher in the oral intake group (median 17% vs. 14%). At long-term follow-up (~3.5 years), median weight loss decreased to 12%, with 35% of patients showing no clinically significant weight loss; long-term outcomes were comparable between oral intake and tube-fed patients (11% vs. 12.5%). Comparative results across time points are summarized in **Table 3** and illustrated in **Figure 1**.

**Table 3:** Weight loss comparison.

Weight loss comparison		
	Oral intake	Feeding tube
Weight loss at the end of radiotherapy		
Median weight loss	11,50%	10,50%
Grade 0	4%	13%
Grade 1	25%	34%
Grade 2	64%	50%
Grade 3	7%	3%
Short-term follow-up (< 90 days radiotherapy)	post-	
Median weight loss	15,50%	13%
Grade 0	9%	10%
Grade 1	15%	23%
Grade 2	63%	54%
Grade 3	13%	13%
Medium-term follow-up (> 90 days post-radiotherapy)	ys	
Median weight loss	17%	14%
Grade 0	9%	15%
Grade 1	16%	19%
Grade 2	34%	40%
Grade 3	41%	26%
Final follow-up (median 3,5 year radiotherapy)	s post-	
Median weight loss	11%	12.5%
Grade 0	40%	31%
Grade 1	9%	10%
Grade 2	30%	35%
Grade 3	21%	24%

Grade 0, <5%; Grade 1, 5 - <10% weight loss; Grade 2, 10 - <20%; Grade 3, >=20%.

At final follow-up, patients with minimal weight loss (<5%) since RT initiation had a significantly reduced risk of overall (−86%, p=0.008) and cancer-specific mortality (p=0.014), whereas grade 2 weight loss was associated with increased overall (HR 2.98, p=0.025) and cancer-specific mortality (HR 3.49, p=0.013).

The results indicated that the difference in weight loss between the two groups was not statistically significant (p = 0.064).

#### Outcomes

**Figure 2:**
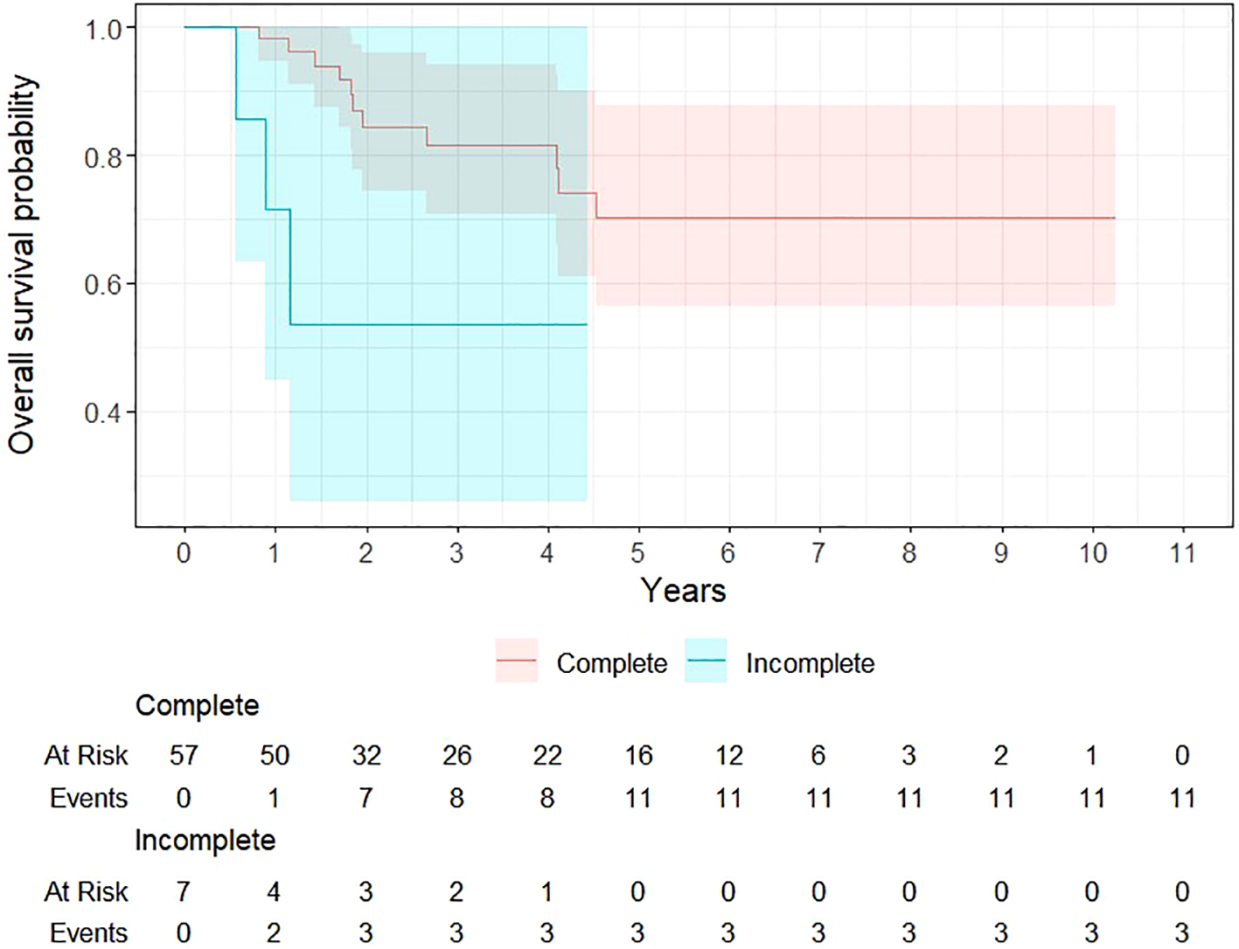
Survival probability based on 3- month FDG-PET-CT nodal response assessment.

In univariable analysis, higher T stage was significantly associated with worse OS, with T4 tumors showing a hazard ratio (HR) of 5.84 (95% CI, 1.58–21.63; p = 0.008). Weight loss during treatment was significantly associated with survival (HR 1.05; 95% CI 1.01–1.09; p = 0.019). Tobacco use showed a trend toward poorer OS for current smokers (HR 2.91; 95% CI 0.97–8.80; p = 0.058). FDG-PET-CT assessment of nodal disease at 3 months post-treatment was significantly associated with OS, with positive nodal findings indicating a higher risk of death (HR 3.75; 95% CI 1.02–13.74; p = 0.046) (**Figure 2**).

Metastatic recurrence (HR 33.11; 95% CI 8.51–128.77; p < 0.001), local recurrence (HR 5.80; 95% CI 2.23–15.08; p < 0.001), and regional recurrence (HR 3.84; 95% CI 1.35–10.91; p = 0.012) were all strongly associated with worse survival. Patients without progression at last follow up had a significantly lower risk of death (HR 0.03; 95% CI 0.01–0.10; p < 0.001).

In multivariable analysis, metastatic recurrence was the strongest independent predictor of death (HR 37.1; 95% CI 7.29–189; p < 0.001), local recurrence showed a trend toward significance (HR 0.26; 95% CI 0.07–1.03; p = 0.055), and status at last follow-up remained highly predictive of survival (HR 0.01; 95% CI 0.00–0.04; p < 0.001). Detailed results of the univariate and multivariate analyses are provided in Annex 2.

**Figure 3:**
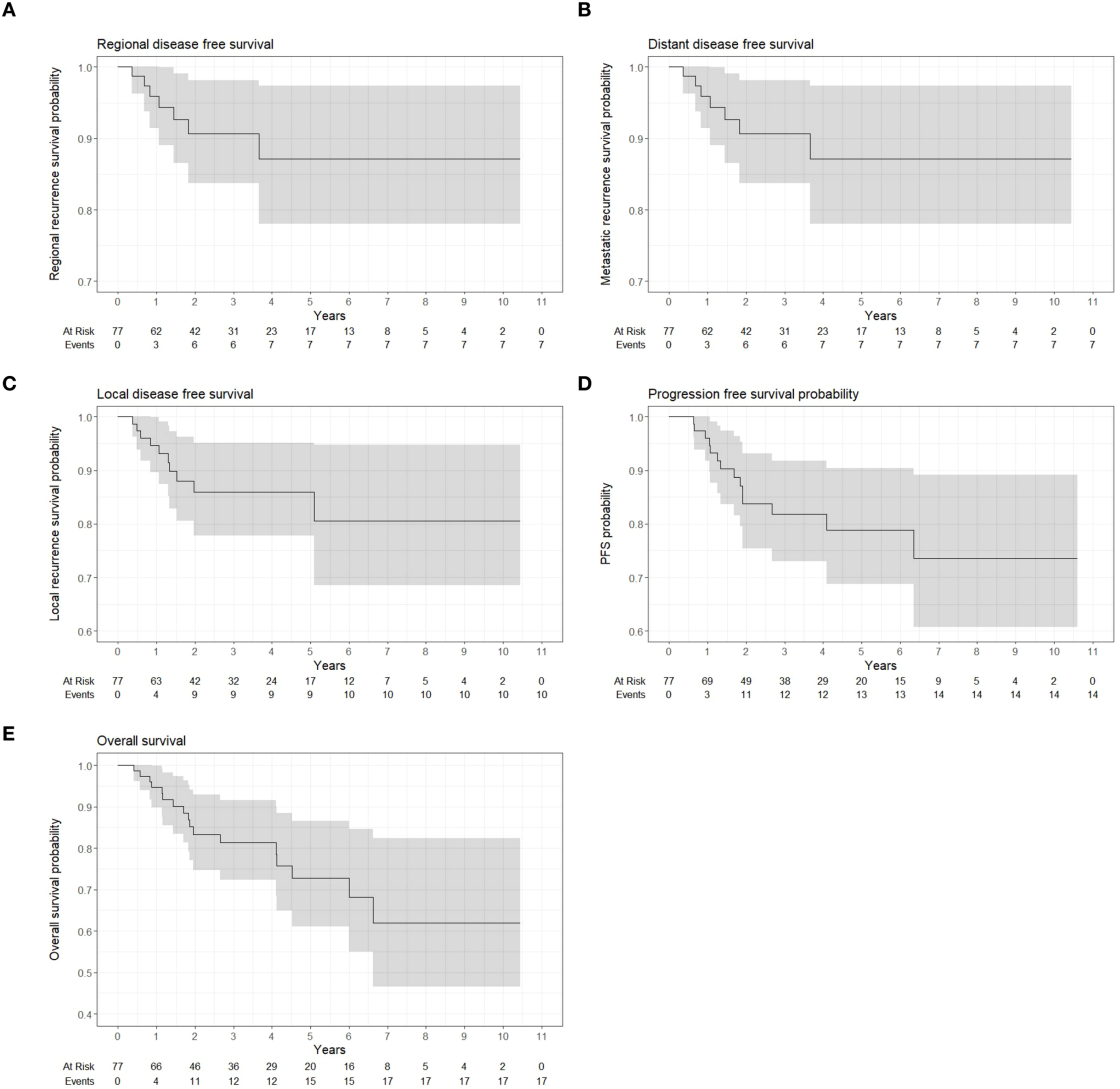
Kaplan-Meier curves showing local disease-free **(A)**, regional-free **(B)**, distant-free **(C)**, progression-free **(D)** and overall **(E)** survival.

The median OS and PFS were not reached. The PFS probability decreases over time but does not go below 50%. At 2 and 5 years, OS was 83.5% and 72.5%, PFS was 83% and 79%, LDFS was 85.5% at both time points, RDFS was 91% and 87%, and DDFS was 91% and 87%, respectively. Kaplan Meier plots for these end points are shown in **Figure 3**.

Twenty-three patients (28%) experienced treatment failure. Local, regional and distant failures and outcomes are described in **Table 4**. Median time to first recurrence (either local, regional of distant) was 34 weeks. Local relapse occurred in 10 patients (12%), with a median time to relapse of 61 weeks (range,19-265). Of these, eight recurrences (80%) were in-field within the high-risk CTV, one (10%) was a marginal failure in the cavernous sinus, and one (10%) was an out-of-field recurrence involving the ethmoid sinus. Among these patients, 30% underwent salvage surgery, 20% received re-irradiation, and 1 patient started systemic therapy. Regional (nodal) recurrence was seen in 7 patients (8.5%), with a median time to relapse of 55 weeks (range, 18-191). Among these, five recurrences occurred within the high-dose CTV and two within the low-dose CTV. Seventy-one percent of these patients underwent salvage surgery as a treatment intervention. Notably, 1 patient had a positive PET scan at 3 months, underwent LN surgery at 4 months, and showed negative nodal pathology. Five patients had metastatic disease at diagnosis. Among the initially non-metastatic patients, 11 developed metastases at a median of 17 weeks (range, 13–52 weeks) after diagnosis. The most frequent site of metastasis was bone metastases in 11 patients.

**Table 4:** Local, regional and distant failures and outcomes.

Local, regional and distant failures and outcomes	number patients (% or IQR)
**Any recurrences**	23 (28%)
**Local recurrence**	10 (12%)
• Median time to local reccurence	61 weeks (43)
• Surgery for local recurrence	
Yes	3 (30%)
No	6 (60%)
Unknown	1 (10%)
• Radiotherapy for local reccurence	
Yes	2 (20%)
No	7 (70%)
Unknown	1 (10%)
• Systemic therapy for local relapse	
Yes	1 (10%)
No	7 (70%)
Unknown	2 (20%)
**Regional (nodal) recurrence**	7 (9%)
Median time to first regional reccurence	55 weeks (46)
Surgery at regional reccurence	5 (71%)
Radiotherapy at regional reccurence	0 (0%)
Systemic therapy at regional reccurence	0 (0%)
**Metastasis**	14 (17%)
Median time to metastasis	38 weeks
Surgery at metastasis	1 (7%)
Radiotherapy at metastasis	10 (71%)
Systemic therapy at metastasis	11 (79%)
**Site metastasis**	14 (17%)
Lung	1 (7%)
Bone	5 (36%)
Liver	1 (7%)
Bone + Liver	3 (21%)
Bone + Lung	2 (14%)
Lung + Liver + Bone	1 (7%)
Axillary lymph nodes	1 (7%)

A total of 22 deaths were recorded, of which 16 were cancer-related. Several clinical factors—including M stage, tumour stage, continued dependence on a feeding tube at short-term follow-up, nodal response on FDG-PET-CT, recurrence (local, regional, or distant), and weight change—were significantly associated with cancer-related mortality.

## Discussion

This cohort study reports outcomes and treatment related toxicity in patients with NPC treated in Western Europe. Although this study was conducted in Belgium, a non-endemic region for NPC, the majority of patients diagnosed (66%) were of Northern African descent, known to be endemic for NPC. This overrepresentation suggests that, despite being diagnosed in a low-incidence country, the epidemiological characteristics of the disease may still be influenced by patients’ geographic and ethnic backgrounds. Detailed data on immigration status or generational history were not systematically available; however, the predominance of Northern African and Asian ancestry suggests that many patients may represent first- or second-generation immigrants. Additionally, a family history of NPC was reported in 7% of our patients, further supporting the hypothesis of a potential genetic predisposition in certain populations. Therefore, even in non-endemic settings like Belgium, the clinical and biological features of NPC may still reflect the risk profile of populations originally from endemic regions, underscoring the importance of considering ethnic and geographic origins in both research and clinical management. Due to the very small number of EBV-negative patients (n=6, 7%), meaningful outcome comparisons between EBV-positive and EBV-negative groups were not feasible. The use of chemotherapy in our cohort was low but comparable in other reported series, with 32% of patients receiving induction chemotherapy and only 6% receiving adjuvant chemotherapy (**Table 5**). This lower utilization may reflect contraindications to cisplatin, including renal impairment, hearing loss, neuropathy, cardiovascular disease, or poor performance status. Most patients had a favorable performance status (WHO 1, 67%), while 33% had WHO >1. However, the retrospective design and incomplete data on comorbidities limited our ability to fully assess their potential impact on treatment decisions and outcomes. Treatment advancements for NPC have substantially improved outcomes have substantially improved outcomes in recent years. Improvements in imaging and treatment planning, including MRI-guided delineation and IMRT, allow precise targeting of the primary tumor and regional lymphatics while sparing critical normal tissues. International consensus guidelines, such as those by Lee et al. (25) have further standardized contouring practices, enhancing consistency across centers and reducing interobserver variability. These evolving practices have, however, contributed to the heterogeneity observed in our patient population. Treatment response was assessed using FDG-PET-CT imaging at three months post-RT to assess early local and nodal tumor responses. This timing is supported by previous research indicating that FDG-PET-CT is a reliable modality for detecting residual disease and differentiating between treatment effects and persistent tumor (27). Studies such as Jeog et al. (33) have demonstrated FDG-PET-CT response to correlate well with long-term outcomes in patients undergoing RT for NPC. Moreover, also the assessment of locoregional nodal response via FDG-PET-CT has been shown to carry significant prognostic value (34). Our study aligns with this body of evidence, reinforcing the critical role of early FDG-PET-CT assessment in post-RT management and prognosis. The ability to early identify patients with suboptimal nodal response may allow for more personalized therapeutic strategies aimed at improving survival outcomes.

**Table 5:** Comparison of our study outcomes with selected series from non-endemic regions.

Reference	n	AJCC stage	Histological subtype, WHO	Treatment	Local control	Regional (nodal) control	Distant control	Overall survival
Present	82*	I 5 (5%), II 19 (23%) III 31 (38%) IVA 23 (28%) IVB 5 (6%)	I 3 (4%), IIa 12 (14%) IIb 33 (40%) Non-keratinising, Diff. missing 34 (42%)	(C)RT ± induction/adjuvant (IMRT) Induction + Concurrent 26 (32%)	5 years 86% *	5 years 87%*	5 years 87%*	5 years 73% *
Setton et al, 2016 (28)	177	I 19 (11%) II 40 (23%) III 72 (41%) IVA 21 (12%) IVB 25 (14%)	I 11 (6%) IIa/IIb 125 (69%) Basaloid 2 (1%) Unknown 39 (21%)	(C)RT ± induction/adjuvant Induction + Concurrent 9 (5%)	3-/5-year 92%/83%	3-/5-year 93%/91%	3-/5-year 86%/83%	3-/5-year: 87%/74%
Slevin et al, 2020 (29)	151	I 4 (3%) II 35 (23%) III 59 (39%) IV 53 (35%)	I 31 (21%) II 52 (34%) III 61 (40%) Unknown 7 (5%)	(C)RT ± induction/adjuvant Induction + Concurrent 82 (54%)	5 years 91%	5 years 94%	5 years 82%	5-year OS 70% 5-year DFS 65%
Bossi et al, 2021 (30)	1230	I-II 246 (20%) III-IVA-IVB 875 (71%) IVC 58 (5%)	Keratinising 146 (12%) Non-keratinising 1051 (86%) Basaloid 18 (1%) Unknown 15 (1%)	(C)RT induction/adjuvant (3DCRT or IMRT) Induction + Concurrent 547 (44%)	Not reported	Not reported	Not reported	5-year OS 84%, n=1021 5-year DFS 65% n=1113
Howlett et al, 2024 (31)	601	I 68 (11.3%) II 198 (32.9%) III 229 (38.1%) IV 106 (17.6%)	I 109 (18%) II 487 (81%) III 5 (1%)	(C)RT ± induction/adjuvant Induction + Concurrent 44 (7%)	5 years Local recurrence 58%	5 years Regional recurrence 22%	5 years Distant recurrence 20%	5-year OS 70%
Alsavaf et al, 2025 (32)	159	I-II 27 (18%) III 53 (35%) IVA/B 70 (47%)	I 15 (11.7%) II 25 (19.5%) III 88 (68.8%) Unknown 31 (1%)	(C)RT ± induction/adjuvant Induction + Concurrent 48 (31%)	Not reported	Not reported	Not reported	12-mo OS: type I 26.7%, type II 76.0%, type III 89.7%

CRT, chemotherapy; n, number of patients.

*Survival analysis were performed excluding 5 patients with metastatic disease at diagnosis.

The observed toxicity profile aligns with existing literature on head and neck RT, confirming that while most acute side effects resolve, several late complications persist and significantly impact on survivorship. Xerostomia remained the most common late effect, consistent with studies showing that despite advances like IMRT, up to 40–60% of patients continue to experience grade ≥2 xerostomia long-term (35–37). Similarly, the high incidence of hypothyroidism mirrors prior findings, with reported rates ranging from 20% to over 50%, particularly when the thyroid receives moderate to high radiation doses (38). Ototoxicity, including tinnitus and subjective hearing loss, increased over time, data supported by Chattaraj et al. (39) who noted delayed onset sensorineural hearing loss, especially in patients receiving cisplatin. The absence of baseline audiometry in our study limits definitive conclusions but reinforces recommendations for pretreatment hearing assessments in similar populations. Although less common, late neurological effects such as cranial nerve impairments and Lhermitte’s sign are clinically important. Lhermitte’s sign, characterized by electric shock sensations along the spine to the limbs upon neck movements, is reported to occur in 1% to 36% of patients following IMRT to the head and neck region (40–42). Symptoms may appear within the first few months post-irradiation of the cervical spinal cord and often resolve spontaneously without leading to permanent or progressive spinal cord damage.

Nutritional status emerged as a key prognostic factor, with mean weight loss reaching 11% by the end of radiotherapy. Weight loss peaked during short- to medium-term follow-up, with grade 3 loss affecting over one-third of patients by 90 days post-treatment. Although definitions of critical weight loss vary across studies, the magnitude and temporal pattern observed in our cohort are broadly consistent with published data reporting a pooled CWL prevalence of 57% during radiotherapy (43). Despite more than half of patients maintaining oral intake, greater weight loss was observed in this group compared with tube-fed patients, suggesting that oral intake alone may be insufficient during intensive treatment. Enteral feeding was associated with modestly improved weight preservation during critical phases but did not fully prevent severe weight loss, underscoring the importance of early identification of high-risk patients, individualized nutritional interventions, and extended post-treatment surveillance. Adaptive replanning during treatment was not systematically assessed in this cohort; therefore, its potential impact on toxicities such as xerostomia, dysphagia, or weight loss could not be evaluated and represents a limitation.

Our study demonstrated favorable outcomes, with actuarial 5-year local, regional, and distant control rates of 86%, 87%, and 87%, respectively, and an estimated 5-year overall survival of 73%, derived from Kaplan–Meier analysis. These results should be interpreted in light of the median follow-up of approximately three years.Setton et al. (2016) (28) reported 5-year local, regional, and distant control rates of 83%, 91%, and 83%, respectively, with a 5-year OS of 74%, in a predominantly non-endemic North American cohort. Similarly, Slevin et al. (2020) (29) observed 5-year local and regional control exceeding 90% and a 5-year OS of 70% in a cohort with a high proportion of advanced-stage disease. Bossi et al. (2021) (30), in a large chort study, reported a 5-year OS of 84% and 5-year DFS of 65%, although detailed local and regional control data were not reported. Howlett et al. (2024) (31) reported somewhat lower local and regional recurrence-free survival (5-year local recurrence 58%, regional 22%) and OS of 70%, reflecting differences in patient selection and follow-up duration. Finally, Alsavaf et al. (2025) (32) highlighted the prognostic impact of histological subtype, with 12-month OS ranging from 27% for type I to nearly 90% for type III NPC (**Table 5**). The patterns of failure observed in this cohort, characterized predominantly by local and regional recurrences occurring in-field high-risk CTV and by early distant metastases, may partly explain the slightly lower-end outcomes.

Limitations of our study include its retrospective design and modest and mixed sample size, potentially constraining the generalizability of our findings.

In conclusion, this study highlights the multifaceted management demands of NPC, advocating for multimodal and intens therapeutic approaches, early treatment response evaluation, and vigilant nutritional support. Our identified prognostic factors offer a framework for tailored treatment strategies aimed at enhancing survival and quality of life in NPC patients. Based on the findings of our series, we propose a structured approach for managing newly diagnosed nasopharyngeal carcinoma at our center. All patients undergo a comprehensive baseline evaluation, including nasopharyngeal endoscopy, MRI of the nasopharynx and neck, FDG-PET/CT, systematic EBV DNA testing and follow-up, and paramedical counseling including dietitian and tobacco cessation support. Induction chemotherapy is considered for all locally advanced cases (18, 44) followed by concurrent intensity-modulated radiotherapy (IMRT) with target volumes delineated according to recent international consensus guidelines (25, 45) consensus guidelines. Peer review of contours involving a dedicated head and neck radiologist and nuclear medicine physician is organized weekly. Treatment planning prioritizes optimal tumor coverage while minimizing dose to critical organs at risk. Response assessment is performed through MRI and FDG-PET/CT, complemented by clinical evaluation via endoscopy and plasma EBV DNA testing (46). Adjuvant chemotherapy may be considered in selected high-risk patients (21, 44). Ongoing research and adherence to evolving clinical standards will be crucial in optimizing outcomes for this complex malignancy.

## Data Availability

The raw data supporting the conclusions of this article will be made available by the authors, without undue reservation.
