# Supplementary material for: Long-term outcomes and patient profiles following intensity-modulated radio-chemotherapy for nasopharyngeal cancer in a nonendemic region
Source: Front Oncol. 2026 Jan 27;16:1724193. doi: 10.3389/fonc.2026.1724193 (PMC12885988; doi:10.3389/fonc.2026.1724193)
Supplement: Supplementary file 1 [file Table1.docx]

Annexe 1. Chemotherapy and number of administered cycles

| **Type of chemotherapy** | **Number of cycles** | **Number of patients** |
| --- | --- | --- |
| Induction | | 26 |
| Cisplatin/Gemcitabine | 2 | 1 |
|  | 3 | 16 |
|  | 4 | 1 |
| Docetaxel/cisplatin/fluorouracil (TPF) | 2 | 1 |
|  | 3 | 5 |
| Cisplatin + 5-FU | 2 | 1 |
|  | 31 |  |
| Concurrent | | 75 |
|  | | |
| Cisplatinum 100mg/m^3^ | 1 | 1 |
|  | 2 | 34 |
|  | 3 | 18 |
|  | unknown | 2 |
| Cisplatinum 40mg/m^3^ | 2 | 2 |
|  | 3 | 3 |
|  | 4 | 2 |
|  | 5 | 3 |
|  | 6 | 2 |
|  | 7 | 1 |
| Carboplanin Weekly | 3 | 1 |
|  | 4 | 1 |
|  | 5 | 1 |
|  | 6 | 3 |
| Cisplatin + 5-FU | 4 | 1 |
| Adjuvant | | 5 |
| Cisplatin + 5-FU | 2 | 1 |
|  | 3 | 2 |
| Carboplatinum + 5 FU | unknown | 1 |
| Capecitabine | 6 | 1 |
