# Supplementary material for: Long-term outcomes and patient profiles following intensity-modulated radio-chemotherapy for nasopharyngeal cancer in a nonendemic region
Source: Front Oncol. 2026 Jan 27;16:1724193. doi: 10.3389/fonc.2026.1724193 (PMC12885988; doi:10.3389/fonc.2026.1724193)
Supplement: Supplementary file 2 [file Table2.docx]

# Overall Survival

Univariable and multivariable survival analyses were performed in a cohort restricted to stage M0 patients (n = 77). During follow-up, 17 deaths occurred and 60 observations were censored.

Given the limited number of events (17 deaths), a maximum of two variables can be included in the multivariable analysis to avoid overfitting.

The significance level was set at p = 0.05.

| Univariate analysis | | | | Multivariate analysis | | |
| --- | --- | --- | --- | --- | --- | --- |
| Predictors | Estimtaes (95% CI) | P-value | Estimates (95% CI) | | P-value |  |
| Race   - Western Europe - Southern Europe - Northern Africa | 2.12 (0.57-7.92)  1.48 (0.42-5.21)  0.86(0.32-2.34) | 0.263  0.544  0.770 |  | |  |  |
| Chemotherapy   - Concomitant - Neo-adjuvant - Adjuvant | 75499646 (0.00-Inf)  1.01 (0.28-3.67)  0.47 (0.06-3.73) | 0.998  0.989  0.479 |  | |  |  |
| Medical background   - Oncological history - Familial history | 0.86 (0.11-6.53)  0.00 (0.00-Inf) | 0.888  0.998 |  | |  |  |
| Alcoholic consumption (ref : Never)   - Current - Former - Occasional | 1.71 (0.37-7.94)  0.00 (0.00-Inf)  1.57 (0.56-4.42) | 0.493  0.998  0.395 |  | |  |  |
| Tabagic consumption (ref : Never)   - Current - Former - Occasional | 2.91 (0.97-8.80)  1.19(0.23-6.15)  3.53 (0.41-30.49) | 0.058  0.837  0.252 |  | |  |  |
| Staging   - T (ref = 1) - T0 - T2 - T3 - T4 - N (ref = 1) - N0 - N2 - N3 | 1. (0.00-Inf)   2.38 (0.48-11.88)  2.28 (0.38-13.67)  5.84 (1.58-21.63)  1.37(0.23-8.22)  2.04 (0.56-7.43)  3.86(0.63-23.90) | 0.998  0.289  0.368  **0.008**  0.729  0.278  0.143 |  | |  |  |
| Stadium (ref = 1,2 )   - 3, 4A, 4B | 6.61 (0.88-49.89) | 0.067 |  | |  |  |
| EBV status (ref = EBV -)   - EBV + | 0.27 (0.06-1.25) | 0.094 |  | |  |  |
| Radiotherapy   - Time between biopsy and radiotherapy - Difference in weight % | 0.99(0.98-1.01)  1.01 (0.93-1.10) | 0.510  0.789 |  | |  |  |
| Nutrition  Type nutrition (ref = oral feeding)   - Parenterale - Feeding tube   Weight loss  Difference in weight from rt  End of radiotherapy   - Grade 0 - Grade 1 - Grade2 - Grade3   Acute toxicity   - Grade 0 - Grade 1 - Grade2 - Grade3   Late toxicity   - Grade 0 - Grade 1 - Grade2 - Grade3   Last follow-up   - Grade 0 - Grade 1 - Grade2 - Grade3 | 1. (0.00-Inf)   1.93 (0.74-5.03)  1.05 (1.01-1.09)   1. (0.00-Inf)   0.66 (0.22-2.04)  2.58(0.84-7.99)  0.00 (0.00-Inf)  1.02 (0.13-7.78)  1.34 (0.38-4.75)  1.51 (0.54-4.19)  0.63 (0.14-2.79)  0.58 (0.08-4.44)  2.03 (0.72-5.71)  1.14 (0.44-2.95)  0.63 (0.20-1.99)  0.16 (0.04-0.70)  1.28 (0.28-5.81)  2.48 (0.86-7.14)  2.19 (0.74-6.46) | 0.998  0.178  **0.019**  0.998  0.474  0.099  0.998  0.986  0.654  0.431  0.541  0.603  0.179  0.793  0.433  **0.015**  0.753  0.092  0.155 |  | |  |  |
| Imaging at 3 months   - CT T - IRM T - IRM N - PET T - PET N | 2.45(0.12-51.96)  1.30 (0.40-4.28)  1.30 (0.40-4.28)  2.08 (0.58-7.37)  3.75 (1.02-13.74) | 0.565  0.665  0.665  0.259  **0.046** |  | |  |  |
| Metastasis recidive (ref : No) | 33.11(8.51-128.77) | **<0.001** |  | |  |  |
| Local recurrence | 5.80 (2.23-15.08) | **<0.001** | 0.26(0.07-1.03) | | **0.055** |  |
| Regional recurrence | 3.84(1.35-10.91) | **0.012** |  | |  |  |
| Status at last follow-up (Ref : Progression) | 0.03 (0.01-0.10) | **<0.001** | 0.01 (0.00-0.04) | | **<0.001** |  |

# Cancer specific survival

For the cancer related death, 13 deaths were attributed to cancer, 4 deaths were not cancer-related, and 60 observations were censored.

With only 13 cancer-related events, a maximum of two variables can be included in the multivariable analysis to avoid overfitting.

The significance level was set at 0.05.

| Univariate analysis | | | | | Multivariate analysis | | | |
| --- | --- | --- | --- | --- | --- | --- | --- | --- |
| Predictors | Estimates HR (95% CI) | P-value | | Estimates (95% CI) | | P-value | |  |
| Race   - Western Europe - Southern Europe - Northern Africa | 2.47 (0.70-8.68)  1.32 (0.28-6.20)  0.78 (0.26-2.35) | 0.200  0.700  0.700 | |  | |  | |  |
| Chemotherapy   - Concomitant - Neo-adjuvant - Adjuvant | 25.37 (9.092-70.23)  0.73 (0.16-3.23)  0.77 (0.17-3.48) | **<0.001**  0.700  0.700 | |  | |  | |  |
| Medical background   - Oncological history - Family history^1^ | 1.18 (0.17-8.28) | 0.900 | |  | |  | |  |
| Alcoholic consumption (ref : Never)   - Current - Former^1^ - Occasional | 2.56 (0.65-10.1)  1.19 (0.59-6.13) | 0.2  0.3 | |  | |  | |  |
| Tabagic consumption (ref : Never)   - Current - Former - Occasional | 3.76 (1.04-13.5)  1.99 (0.35-11.3)  6.87 (0.71-66.2) | **0.043**  0.400  0.10 | |  | |  | |  |
| Staging   - T (ref = 1) - T0^2^ - T2 - T3 - T4 - N (ref = 1) - N0^2^ - N2 - N3 | 1.5 (0.24-9.39)  2.18 (0.42-11.3)  3.38 (0.85-13.4)  2.82 (0.65-12.3)  4.76 (0.77-29.6) | 0.70  0.40  0.08  0.200  0.094 | |  | |  | |  |
| Stadium (ref = 1,2)   - III, IVA | 38.794 (19.14-78.63) | **<0.001** | | 6.01 (1.85-19.6) | | **<0.001** | |  |
| EBV status (ref = EBV -)   - EBV + | 0.44 (0.05-3.63) | 0.400 | |  | |  | |  |
| Radiotherapy   - Time between biopsy and radiotherapy - Difference in weight % | 1. (0.98-1.01)   0.99(0.97-1.01) | 0.600  0.200 | |  | |  | |  |
| Nutrition  Type nutrition (ref = oral feeding)   - Parenterale^1^ - Feeding tube   Weight loss  Difference in weight from rt  End of radiotherapy   - Grade 0^2^ - Grade 1 - Grade2 - Grade3^2^   Acute toxicity   - Grade 0 - Grade 1 - Grade2 - Grade3   Late toxicity   - Grade 0 - Grade 1 - Grade2 - Grade3   Last follow-up   - Grade 0 - Grade 1 - Grade2 - Grade3 | 1.87 (0.65-5.44)  1.04 (1.01-1.07)  0.95 (0.31-2.94)  1.87 (0.59-5.93)  1.25 (0.18-8.76)  1.58 (0.45-5.53)  0.74 (0.25-2.17)  0.92 (0.21-4.07)  0.78 (0.10-5.83)  1.08 (0.22-5.29)  1.16 (0.38-3.60)  0.89(0.27-2.95)  0.09 (0.01-0.71)  1.59 (0.41-6.09)  2.95 (0.97-8.96)  1.83 (0.57-5.87) | 0.300  **0.004**  >0.900  0.300  0.800  0.500  0.600  >0.900  0.800  >0.900  0.800  0.900  **0.022**  0.500  0.057  0.300 | |  | |  | |  |
| Imaging at 3 months (ref: complete)   - CT T - IRM T - IRM N - PET T - PET N | 2.45 (0.41-14.8)  1.26 (0.32-4.86)  1.26 (0.32-4.86)  1.69 (0.43-6.66)  4.02 (0.96-16.7) | 0.300  0.700  0.700  0.500  0.056 | |  | |  | |  |
| Metastasis recidive (ref : No) | 54.7 (11.0-272) | **<0.001** | | 37.1 (7.29-189) | | **<0.001** | |  |
| Local recurrence | 10.7 (3.78-30.4) | **<0.001** | |  | |  | |  |
| Regional recurrence | 4.0 (1.17-8.71) | **0.009** | |  | |  | |  |
| Status at last follow-up (Ref: Progression)^1^ |  |  | |  | |  | |  |
| ^1^: Very small number of patients with the same outcome | | |  | |  | |  | |
